# Increased Production of the Value-Added Biopolymers Poly(*R*-3-Hydroxyalkanoate) and Poly(γ-Glutamic Acid) From Hydrolyzed Paper Recycling Waste Fines

**DOI:** 10.3389/fbioe.2019.00409

**Published:** 2019-12-18

**Authors:** Ryan A. Scheel, Alexander D. Fusi, Byeong C. Min, Christopher M. Thomas, Bandaru V. Ramarao, Christopher T. Nomura

**Affiliations:** ^1^Department of Chemistry, State University of New York College of Environmental Science and Forestry, Syracuse, NY, United States; ^2^Department of Paper and Bioprocess Engineering, State University of New York College of Environmental Science and Forestry, Syracuse, NY, United States; ^3^Center for Applied Microbiology, State University of New York College of Environmental Science and Forestry, Syracuse, NY, United States

**Keywords:** biopolymer, polyhydroxyalkanoates, polyglutamic acid, biosynthesis, lignocellulosic, waste stream, linerboard recycling

## Abstract

Reject fines, a waste stream of short lignocellulosic fibers produced from paper linerboard recycling, are a cellulose-rich paper mill byproduct that can be hydrolyzed enzymatically into fermentable sugars. In this study, the use of hydrolyzed reject fines as a carbon source for bacterial biosynthesis of poly(*R*-3-hydroxyalkanoate) (PHA) and poly(γ-glutamic acid) (PGA) was investigated. Recombinant *Escherichia coli* harboring PHA biosynthesis genes were cultivated with purified sugars or crude hydrolysate to produce both poly(*R*-3-hydroxybutyrate) (PHB) homopolymer and medium chain length-containing copolymer (PHB-*co*-MCL). Wild-type *Bacillus licheniformis* WX-02 were cultivated with crude hydrolysate to produce PGA. Both PHB and short chain-length-*co*-medium chain-length (SCL-*co*-MCL) PHA yields from crude hydrolysate were a 2-fold improvement over purified sugars, and the MCL monomer fraction was decreased slightly in copolymers produced from crude hydrolysate. PGA yield from crude hydrolysate was similarly increased 2-fold. The results suggest that sugars from hydrolyzed reject fines are a viable carbon source for PHA and PGA biosynthesis. The use of crude hydrolysate is not only possible but beneficial for biopolymer production, eliminating the need for costly separation and purification techniques. This study demonstrates the potential to divert a lignocellulosic waste stream into valuable biomaterials, mitigating the environmental impacts of solid waste disposal.

## Introduction

Paper waste fines are cellulose fibers that have become too short for incorporation into paper products due to repeated recycling and must therefore be rejected from this process. These rejected waste fines make up a significant proportion of the waste stream from paper mills, which is becoming increasingly difficult to landfill due to transportation costs and legislation (Villanueva and Wenzel, 2007; Laurijssen et al., 2010). Since reject fines are predominantly composed of cellulose they can be readily hydrolyzed into monomeric sugars making them an attractive waste stream for the production of value-added products, including biofuels, platform chemicals, and biopolymers such as polyhydroxyalkanoates (Galbe and Zacchi, 2002; Zhang, 2008; Wang et al., 2013; Bhuwal et al., 2014; Min et al., 2015). Waste fines from the recycling of old corrugated cartons (OCC) are particularly valuable as a source of fermentable sugars, as they are typically high in cellulose and low in lignin and other inhibitory chemicals and minerals compared with deinked paper pulp from other waste streams (Min et al., 2015; Saini et al., 2019).

Polyhydroxyalkanoates (PHAs) are a diverse class of bacterially produced polyesters known for their biodegradability and biocompatibility, which occur naturally as a form of carbon storage (Lee, 1996; Lu et al., 2009). The physical characteristics of PHAs are dependent on both monomeric composition and molecular weight, and they range from stiff and brittle crystalline materials to flexible and elastomeric amorphous polymers (Laycock et al., 2013). Poly(*R*-3-hydroxybutyrate) (PHB) is the most abundant PHA from both natural and anthropogenic sources; however, this material is of limited use due to its high brittleness. The copolymerization of 3HB with other monomers, particularly those of medium chain-length (6–14 carbons), can improve toughness and elasticity for a more versatile material (Noda et al., 2005). The large-scale production and utilization of PHAs is mainly limited by the production cost, a large portion of which stems from the cost of the feedstock and which is high relative to the production costs for petroleum-based plastics with similar properties. One way to address these costs is to examine alternative inexpensive feedstocks, which has sparked interest in lignocellulosic waste streams as a cheap carbon source.

Poly (γ-glutamic acid) (PGA) is another biopolymer that has generated interest as a renewable material for a number of applications. PGA is biosynthesized naturally by a variety of *Bacillus* species, and is an edible, water-soluble, biodegradable, and anionic biopolymer (Bajaj and Singhal, 2011; Ogunleye et al., 2015). These properties make PGA suited for a variety of applications, including metal-ion binding and flocculation for wastewater treatment, composite materials for tissue engineering and drug delivery, and as a medicinal metal chelator for heavy metal removal (Yokoi et al., 1996; Shih et al., 2001; Ye et al., 2006; Siao et al., 2009; Inbaraj and Chen, 2012). Current research into improving PGA production for human use is focused on the metabolic engineering of various *Bacillus* species; notably, wild-type *B. licheniformis* WX-02 is capable of producing large amounts of PGA from glucose and glutamate and has been successfully engineered for enhanced biosynthesis (Cai et al., 2017, 2018).

The work presented in this study demonstrates the successful biosynthesis of both PHA and PGA biopolymers from crude hydrolyzed paper waste fines.

## Materials and Methods

### Hydrolysate From Waste Fines

A recycled liner board mill provided waste fines from the screw-press sludge. The analysis of the waste fines is provided in Min et al. (2018). The enzymatic hydrolysis procedure was conducted as described by Min and Ramarao (2017). Enzymatic hydrolysis was conducted at 50°C and with commercially available CTec2 enzymes (Novozymes USA) at a substrate consistency of 5%. All other details are described by Min et al. (2018) and Min and Ramarao (2017).

### Media and Cultivation

A complete list of strains and plasmids is shown in Table 1. All *E. coli* strains were grown on LB-Lennox (LB; composition per liter: 10 g tryptone, 5 g yeast extract, and 5 g sodium chloride, pH 7.0) purchased from Difco, with 15 g L^−1^ agar when needed. Glucose (Acros Organics) and xylose (Sigma Aldrich) were supplemented as carbon sources when noted, as well as purified or crude linerboard waste fines hydrolysate (Min et al., 2015). *Bacillus licheniformis* WX-02 were maintained using nutrient broth no. 2 (Oxoid) media. For PGA biosynthesis, *B. licheniformis* WX-02 was cultivated in the following PGA biosynthesis media (composition per liter): 90 g glucose (or 10% crude hydrolysate, v/v), 40 g sodium glutamate, 10 g sodium citrate trihydrate, 10 g sodium nitrate, 8 g ammonium chloride, 1 g potassium phosphate trihydrate, 1 g magnesium sulfate heptahydrate, 1 g zinc sulfate heptahydrate, 1 g calcium chloride, 0.15 g manganese sulfate monohydrate, pH 7.3 ± 0.1. *E. coli* strains were made chemically competent and transformed by heat shock following standard procedures (Sambrook and Russell, 2001), and selection was performed on LB agar (15 g L^−1^) plates. The antibiotics kanamycin (50 mg L^−1^) and ampicillin (100 mg L^−1^) were added to media for selection and plasmid retention as appropriate. All liquid media cultures were cultivated using a rotary shaking incubator (New Brunswick Scientific).

**Table 1:** Bacterial strains and plasmids.

**Strains/Plasmids**	**Relevant characteristics**	**References**
BW25113	*E. coli, Δ(araBAD)567, ΔlacZ4787(::rrnB3), λ^−^, rph-1, Δ(rhaBAD)568, hsdR514*	Lessard et al., 1998; Datsenko and Wanner, 2000
LSBJ	*E. coli* LS5218, *ΔfadB, ΔfadJ, atoC512* (Const), *fadR601*	Tappel et al., 2012
*B. licheniformis*	*Bacillus licheniformis* WX-02, saline soil isolate.	Wei et al., 2010
pBBRSTQKAB	pBBR1MCS-2 derivative (lac promoter); *phaC1*(STQK), *phaA, phaB*	Nomura et al., 2004
pTrcGK	pTrc99a derivative (trc promoter); *phaG, alkK*	Wang et al., 2012

PHA biosynthesis methods were adapted from previous studies (Wang et al., 2012; Tappel et al., 2014). Individual colonies of transformed bacteria harboring PHA biosynthesis genes (pBBRSTQKAB or pBBRSTQKAB/pTrcGK) were used to inoculate separate 2 mL LB seed cultures, in triplicate for each strain. Seed cultures were grown for 16 h at 37°C and 200 rpm and used to inoculate 100 mL of LB media in 500-mL baffled shake flasks (final concentration of 0.5%). Shake flasks were cultivated at 30°C and 250 rpm rotary shaking for a total of 48 hrs. After reaching an OD_600_ of 1.0, cultures were induced with isopropyl-β-D-thiogalactoside (IPTG) at a final concentration of 1 mM. Carbon supplements (glucose, xylose, or pure hydrolysate at 20 g L^−1^; or crude hydrolysate at 8% v/v) were added 3 h post-IPTG induction. Cells were collected by centrifugation at 3,716 × g for 15 min, washed once with 45 mL of 35% ethanol and once with 45 mL of water, and dried via lyophilization.

PGA biosynthesis methods were adapted from a previous study (Cai et al., 2018). Individual colonies were used to inoculate separate 2 mL LB seed cultures, in triplicate, and incubated for 12 h at 37°C and 200 rpm. Seed cultures were used to inoculate 100 mL of PGA biosynthesis media in 500-mL baffled shake flasks (final concentration of 0.5%). Shake flasks were cultivated at 37°C and 200 rpm for a total of 36 h. To collect PGA, the pH of 10 mL of culture was adjusted to 2.0 using HCl (conc.) and centrifuged at 8,000 × g for 10 min. The supernatant was collected and neutralized with NaOH (10M), then precipitated with 30 mL of 100% ethanol and mixed by vortex. The precipitate was collected by centrifugation at 8,000 × g for 10 min, then dried via lyophilization to obtain a dry weight.

### Analytical Procedures

The sugar content of the crude hydrolysate was determined using ^1^H-NMR (Kiemle et al., 2003). A calibration curve was generated from the integration of α anomeric proton peaks of pure glucose and xylose at known concentrations relative to glucosamine added as an internal standard (5 g L^−1^). Crude hydrolysate was diluted 10-fold, doped with the glucosamine standard, and the sugar concentrations calculated from the calibration curve. Inductively coupled plasma optical emission spectroscopy (ICP-OES) was used to analyze abundance of metal ions (Optima 5300 DV). Hydrolysate was diluted 10-fold and introduced to the ICP-OES instrument at a flow rate of 1.5 mL min^−1^. Al, As, Ba, Cd, Co, Cu, Cr, Fe, Mo, P, Pb, S, and Zn were analyzed with an axial plasma view, while Ca, K, Mn, Mg, and Na were analyzed radially. Abundance was analyzed by measuring peak areas for each element compared to 4-point calibration curves of known standards.

The yields and repeating unit compositions of PHA polymers were determined using GC, as previously described with slight modification (Braunegg et al., 1978; Scheel et al., 2016). ACS HPLC-grade chloroform and methanol were used for gas chromatography (GC) sample preparation. Lyophilized cells (15–20 mg) were suspended in 2 mL of a 15% (v/v) sulfuric acid solution in methanol and 2 mL of chloroform and heated at 100°C for 140 min in a 10 mL pressure vial (Kimax). The samples were cooled to room temperature, and 1 mL of Nanopure filtered water and 500 μL of methyl octanoate standard (0.25% v/v) in chloroform were added and mixed by vortex. Aqueous and organic layers were separated by centrifugation for 5 min at 700 rpm (Marathon 6K, Fisher Scientific). The organic layer was passed through a 0.2 μm polytetrafluoroethylene (PTFE) filter using a vacuum manifold (Millex Samplicity) into 2 mL GC vials. Samples were injected and separated using a GC 2010 Gas Chromatograph with an AOC-20i autoinjector and a flame ionization detector. Shimadzu's GCSolution software was used to analyze the data. Statistical analyses were performed using the Data Analysis Toolpak for Microsoft Excel.

PGA was verified by ^1^H-NMR spectroscopy using a Bruker AVANCE III 600 MHz instrument. Spectra were processed with Bruker TopSpin v3.5pI2.

## Results

### Hydrolysate Characterization

The concentration of glucose and xylose in the crude linerboard waste fines hydrolysate were calculated to be 98.5 and 28.8 g L^−1^, respectively (Supplementary Figure 1). The metal ion composition of the crude hydrolysate was determined to be: Ca (5486 ppm), Na (1853 ppm), S (21.0 ppm), Mg (20.0 ppm), K (13.0 ppm), Zn (4.70 ppm), Mn (4.44 ppm), Al (2.24 ppm), Ba (1.56 ppm), P (1.12 ppm), Fe (0.30 ppm), Pb (0.24 ppm), Cu (0.19 ppm), Co (40 ppb), As (32 ppb), Mo (17 ppb), Cd (10 ppb), and Cr (7 ppb).

### PHA Analysis

**Figure 1:**
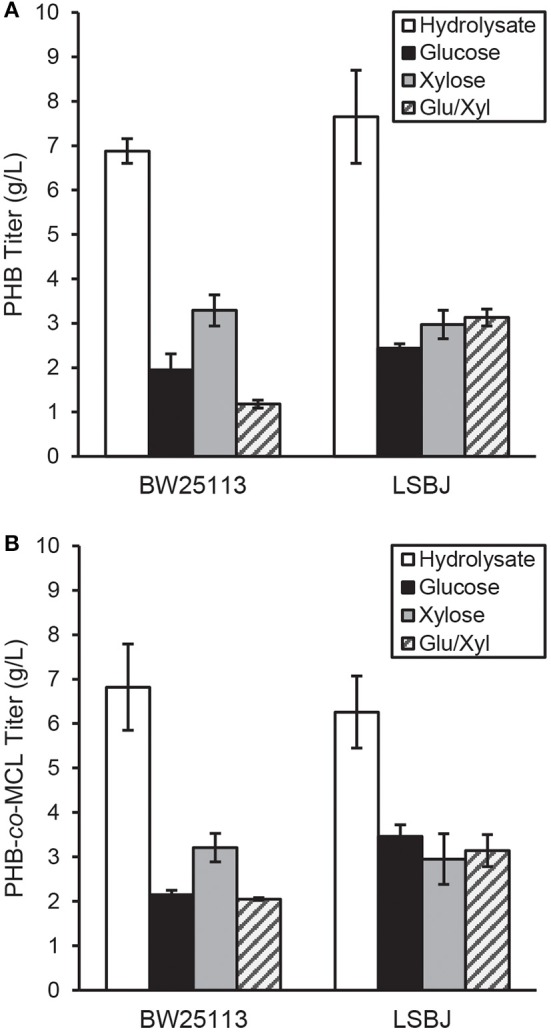
PHA produced by *E. coli* strains BW25113 and LSBJ harboring **(A)** pBBRSTQKAB or **(B)** pBBRSTQKAB and pTrcGK. Yields of PHB in g L^−1^ for each carbon source are shown for both strains in **(A)**, while yields of PHB-*co*-MCL are shown in **(B)**. Data shown are averages and standard deviations of 3 biological replicates. Hydrolysate denotes the crude hydrolysate (white bar), Glu/Xyl denotes purified hydrolysate sugars (gray striped bar), and Glucose and Xylose are store-bought pure sugars (black and solid gray bars, respectively).

PHB and SCL-*co*-MCL PHA were synthesized in recombinant BW25113 and LSBJ using several different carbon sources. The yield of PHB biosynthesized by BW25113 and LSBJ when crude hydrolysate was the carbon source was 6.88 and 7.65 g L^−1^, respectively, which was greater than a 2-fold increase compared with any other pure carbon source analyzed (Figure 1A). These results were determined to be statistically significant using a Student's *T*-test (two-tailed, α = 0.05) comparing yields from each pure carbon source to that of the hydrolysate. PHB yields from pure carbon sources were not significantly different between BW25113 and LSBJ, except for the mixed glucose/xylose which led to yields of 1.18 and 3.13 g L^−1^, respectively.

The yields of SCL-*co-*MCL biosynthesized by BW25113 and LSBJ displayed similar trends to PHB homopolymer production; with crude hydrolysate, PHA yields were 6.82 and 6.26 g L^−1^, respectively, which was slightly less than a 2-fold increase compared to the other carbon sources (Figure 1B). These results were determined to be statistically significant using a Student's *T*-test (two-tailed, α = 0.05) comparing yields from each pure carbon source to that of the hydrolysate. In LSBJ, none of the SCL-*co*-MCL yields from pure carbons were significantly different. However, in BW25113 the utilization of xylose led to significantly higher yields (3.21 g L^−1^) compared to glucose and the mixed glucose/xylose (Figure 1B).

**Figure 2:**
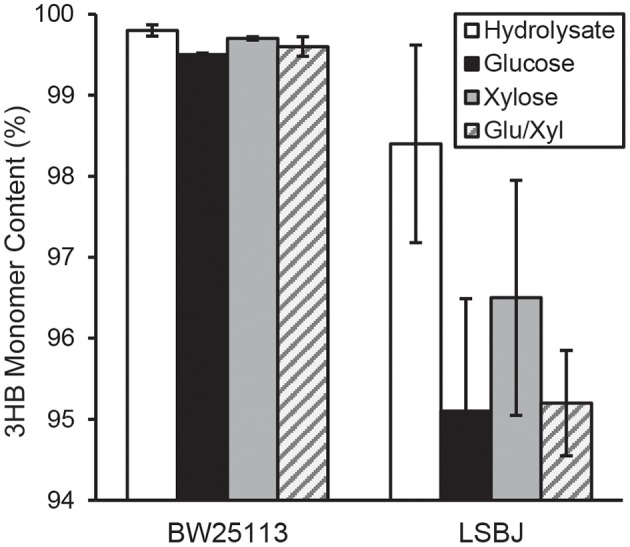
3HB monomer content of PHB-*co*-MCL produced by *E. coli* strains BW25113 and LSBJ harboring pBBRSTQKAB and pTrcGK. Data shown are averages and standard deviations of 3 biological replicates. Hydrolysate denotes the crude hydrolysate (white bar), Glu/Xyl denotes purified hydrolysate sugars (gray striped bar), and Glucose and Xylose are store-bought pure sugars (black and solid gray bars, respectively).

**Figure 3:**
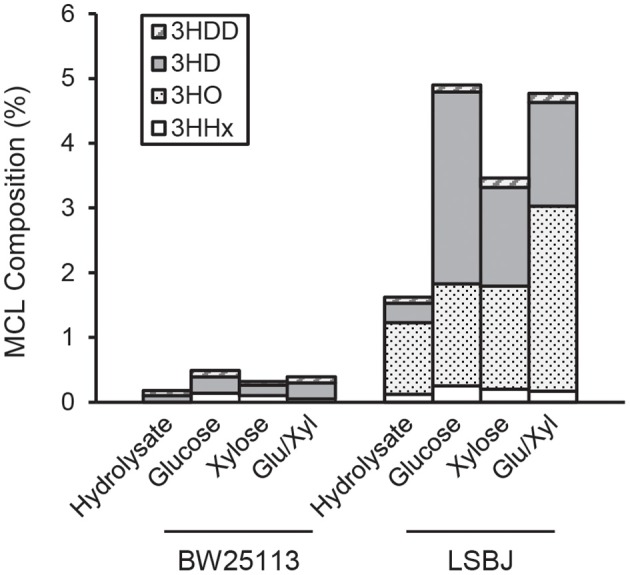
MCL monomer content of PHB-*co*-MCL produced by *E. coli* strains BW25113 and LSBJ harboring pBBRSTQKAB and pTrcGK. No 3HO monomers were detected in polymer produced by BW25113. 3HDD, 3-hydroxydodecanoate (gray striped bar); 3HD, 3-hydroxydecanoate (solid gray bar); 3HO, 3-hydroxyoctanoate (dotted bar); 3HHx, 3-hydroxyhexanoate (white bar).

The 3HB monomer fractions were substantially different between BW25113 and LSBJ, with the former strain producing copolymers with >99.5% 3HB regardless of carbon source (Figure 2). LSBJ produced copolymers with greater variability in their monomer content, though still dominated by 3HB monomers which ranged from 95.1 to 98.4% (Figure 2). Due to the high variation observed in copolymer composition from LSBJ, only PHAs produced from glucose and the mixed glucose/xylose were found to be significantly different between LSBJ and BW25113 using a Student's *T*-test (two-tailed, α = 0.05). The MCL monomer composition followed similar trends between carbon sources, but not between strains; polymers produced by BW25113 contained no observable 3-hydroxyoctanoate (3HO) monomers, whereas 3HO constituted a significant percentage of the MCL fraction of PHA produced by LSBJ (Figure 3). 3-hydroxydecanoate (3HD) was the other dominant MCL monomer in PHA produced by LSBJ, with the combined 3HO and 3HD fractions making up >87% of the total MCL fraction. The MCL monomer compositions were compared between each pure carbon source and the hydrolysate separately for both LSBJ and BW25113 using a two-factor ANOVA with replication (α = 0.05), and each comparison was found to be statistically significant with the exception of xylose from the LSBJ strain.

### PGA Analysis

PGA was synthesized by *Bacillus licheniformis* WX-02 from either pure glucose or the crude hydrolysate. PGA yields from these two carbon supplements were 3.25 and 6.46 g L^−1^ (standard deviations of 0.32 and 0.90), respectively, and were determined to be significantly different using a Student's *T*-test (two-tailed, α = 0.05). The identity of the PGA was confirmed by ^1^H-NMR (Supplementary Figure 2). A minor impurity was observed in the 1H-NMR spectrum (3.08–2.88 ppm), which was confirmed to be unconnected to the polymer backbone by COSY-NMR (Supplementary Figure 3).

## Discussion

In this study there was a marked increase in both PHA and PGA biopolymer yields when substituting the crude hydrolysate for the pure sugar carbon sources. In comparison with previous studies, PHB and SCL-co-MCL PHA yields from crude hydrolysate were significantly improved. Using similar methodologies and an unrelated *E. coli* strain (JM109), Nomura et al. achieved a yield of 2.31 g L^−1^ of PHB from pure glucose (Nomura et al., 2004). Similarly, SCL-co-MCL polymers were previously produced with a yield of 3.49 g L^−1^ using the same growth conditions and *E. coli* LS5218, the parental strain of LSBJ (Tappel et al., 2014). For the PGA production experiment, our results are an improvement over early studies that use similar media and growth methods, where researchers observed a yield of 2.16 g L^−1^ (Wei et al., 2010). However, there have been much greater improvements in yield by optimizing media formulations and genetically modifying WX-02 to improve ATP supply, with recent yields as high as 43.81 g L^−1^ (Wei et al., 2010; Cai et al., 2017, 2018).

Due to the complex composition of the crude hydrolysate, it is difficult to pinpoint and investigate specific hypotheses for the observed increase in biopolymer yield. One possible explanation for these results is the abundance of several important metal ions in the hydrolysate. The most abundant metal in the hydrolysate was calcium which was present at physiologically relevant concentrations (~10 mM in hydrolysate supplemented shake flasks) (Holland et al., 1999). Although the role of calcium in prokaryotes is not completely understood, it has been implicated in processes such as cell division, chemotaxis, and regulation of mechanosensitive ion channels (Norris et al., 1996; Kung et al., 2010; Martins et al., 2011; Booth, 2014; Domínguez et al., 2015). Interestingly, there is also evidence of non-proteinaceous PHB and polyphosphate acting as voltage-gated calcium channels in *E. coli* as a strategy to maintain calcium homeostasis, and the increased PHB yield observed in this study could be partially attributed to that (Reusch et al., 1995; Das et al., 1997). However, the extraction and analysis methods in this study cannot differentiate between transmembrane PHB and intracellular granules, so this is merely speculation.

Mg^2+^ and K^+^ are both vital to bacterial survival, and the presence of these two ions in the hydrolysate may have enhanced bacterial growth (Romani and Scarpa, 2000; Epstein, 2003). A recent study found evidence that peptide-based media may be Mg^2+^-limited, and that *E. coli* grown on tryptone-based media supplemented with glucose were unable to completely utilize that glucose unless supplemented with Mg^2+^(Christensen et al., 2017). However, this would not explain the enhancement of PGA biosynthesis which was carried out in a defined medium with abundant Mg^2+^. Transition metal cofactors such as Fe, Zn, Mn, and Cu are also beneficial for bacterial growth in modest concentrations, and the trace amounts present in the hydrolysate could have also contributed to improved biomass and biopolymer yields (Hood and Skaar, 2012). Although heavy metals such as Pb, As, and Cd are toxic to bacteria, they were not present in high enough concentrations to inhibit bacterial growth (Mitra et al., 1975; Peng et al., 2007; Neumann and Leimkühler, 2008).

The concentration of glucose and other sugars in bacterial media is an important parameter to consider, and under-feeding or over-feeding can significantly alter metabolism (Stephanopoulos et al., 1998). Although 20 g L^−1^ of glucose is often used in PHA experiments, there is evidence that this concentration is above the optimum for sustained bacterial growth (Shang et al., 2003; Christensen et al., 2017). In fed-batch cultures of *Ralstonia eutropha*, PHB yields were highest when glucose was maintained at 9 g L^−1^ and a decrease in biomass and PHB yield was observed as glucose concentration increased (Shang et al., 2003). Other studies have found evidence that carbon or nitrogen limitation can increase PHB yields in recombinant *E. coli* (Wang et al., 2009). This may have contributed to the increased biopolymer yields observed in this study, as the crude hydrolysate was supplemented at lower sugar concentrations than pure sugars (7.30 g L^−1^ glucose and 2.13 g L^−1^ xylose for PHA experiments, 8.95 g L^−1^ glucose and 2.62 g L^−1^ xylose for PGA experiments). Additionally, acetate was present in the hydrolysate at 1 g L^−1^ and has been previously shown to be utilized by *E. coli* LS5218 derivatives and incorporated into PHB (Salamanca-Cardona et al., 2014, 2016). However, this amount of acetate would only contribute a maximum theoretical yield of 0.865 g L^−1^ PHB, which does not account for the total increase of PHB in cells grown on hydrolysate as opposed to pure sugars (Figure 1).

The copolymerization of PHB with MCL 3HA monomers often gives desirable material properties by reducing the crystallinity and brittleness of the resulting polymers, even with MCL fractions as low as 6 mol% (Matsusaki et al., 2000; Sudesh et al., 2000). The SCL-*co*-MCL PHAs produced by LSBJ in this study only incorporated a small amount of MCL monomers, and almost no MCL monomers were incorporated by BW25113 regardless of the carbon source (Figures 2, 3). There are several potential reasons for the low MCL incorporation; these MCL monomers are scavenged from fatty acid biosynthesis by the transacylase activity of PhaG, a putative 3-hydoxyacyl-ACP-CoA acyltransferase (Fiedler et al., 2000; Rehm et al., 2001). Free 3-hydroxy fatty acids can also be incorporated through the 3-hydroxyacyl-CoA ligase activity of AlkK (Wang et al., 2012). The reduced concentration of sugars from crude hydrolysate is likely to slow the growth rate of the cultures and shift metabolism away from fatty acid biosynthesis, resulting in less availability of 3-hydroxyacyl compounds (Takamura and Nomura, 1988; Li and Cronan, 1993). In *E. coli* LSBJ, the transcriptional regulator FadR, which both activates fatty acid biosynthesis and represses β-oxidation, is non-functioning (DiRusso et al., 1992; Iram and Cronan, 2005). This may explain why LSBJ was able to incorporate a greater percent of MCL monomers than BW25113, which has a fully functioning fatty acid biosynthesis pathway and is less likely to have large pools of 3-hydroxyacyl-ACP available.

In this study we have successfully biosynthesized PHB, SCL-*co*-MCL PHA, and PGA using sugars derived from the hydrolysis of linerboard recycling waste fines. Although sugars obtained from this hydrolysis can be purified and do indeed result in PHA biosynthesis, this can be a costly and time-consuming process and generates a low-concentration sugar solution that must be concentrated for bacterial fermentation. The work here has shown that the crude waste fines hydrolysate is a good source of lignocellulosic sugars for value-added biopolymer production.

## Data Availability Statement

The datasets generated for this study are available on request to the corresponding author.

## Author Contributions

CN, BR, and RS conceived and planned the experiments. RS wrote the manuscript with support by CN and BR. The hydrolysates were prepared and analyzed for their compositions by BM with the assistance of CT. RS conducted the majority of the experiments and analyses in this work. AF assisted with copolymer and poly(γ-glutamate) experiments with supervision by RS. All authors contributed to manuscript revision, read, and approved the submitted version.

### Conflict of Interest

Avatar Sustainable Technologies LLC, Syracuse NY, had an option to license technology to produce hydrolysates from waste fines. BR is a co-founder of this company. The remaining authors declare that the research was conducted in the absence of any commercial or financial relationships that could be construed as a potential conflict of interest.

## References

[B1] Bajaj I. Singhal R. (2011). Poly (glutamic acid) – an emerging biopolymer of commercial interest. Bioresour. Technol. 102, 5551–5561. 10.1016/j.biortech.2011.02.047 21377358

[B2] Bhuwal A. K. Singh G. Aggarwal N. K. Goyal V. Yadav A. (2014). Poly-β-hydroxybutyrate production and management of cardboard industry effluent by new *Bacillus* sp. NA10. Bioresour. Bioprocess. 1:9 10.1186/s40643-014-0009-5

[B3] Booth I. R. (2014). Bacterial mechanosensitive channels: progress towards an understanding of their roles in cell physiology. Curr. Opin. Microbiol. 18, 16–22. 10.1016/j.mib.2014.01.005 24607989 PMC4005912

[B4] Braunegg G. Sonnleitner B. Lafferty R. M. (1978). A rapid gas chromatographic method for the determination of poly-β-hydroxybutyric acid in microbial biomass. Eur. J. Appl. Microb. Biotechnol. 6, 29–37. 10.1007/BF00500854

[B5] Cai D. Chen Y. He P. Wang S. Mo F. Li X.. (2018). Enhanced production of poly-γ-glutamic acid by improving ATP supply in metabolically engineered *Bacillus licheniformis*. Biotechnol. Bioeng. 115, 2541–2553. 10.1002/bit.26774 29940069

[B6] Cai D. He P. Lu X. Zhu C. Zhu J. Zhan Y.. (2017). A novel approach to improve poly-γ-glutamic acid production by NADPH Regeneration in *Bacillus licheniformis* WX-02. Sci. Rep. 7:43404. 10.1038/srep43404 28230096 PMC5322528

[B7] Christensen D. G. Orr J. S. Rao C. V. Wolfe A. J. (2017). Increasing growth yield and decreasing acetylation in *Escherichia coli* by optimizing the carbon-to-magnesium ratio in peptide-based media. Appl. Environ. Microbiol. 83, e03034–e03016. 10.1128/AEM.03034-16 28062462 PMC5335519

[B8] Das S. Lengweiler U. D. Seebach D. Reusch R. N. (1997). Proof for a nonproteinaceous calcium-selective channel in *Escherichia coli* by total synthesis from (*R*)-3-hydroxybutanoic acid and inorganic polyphosphate. Proc. Natl. Acad. Sci. U.S.A. 94, 9075–9079. 10.1073/pnas.94.17.9075 9256437 PMC23036

[B9] Datsenko K. A. Wanner B. L. (2000). One-step inactivation of chromosomal genes in *Escherichia coli* K-12 using PCR products. Proc. Natl. Acad. Sci. U.S.A. 97, 6640–6645. 10.1073/pnas.120163297 10829079 PMC18686

[B10] DiRusso C. C. Heimert T. L. Metzger A. K. (1992). Characterization of FadR, a global transcriptional regulator of fatty acid metabolism in *Escherichia coli*. interaction with the *fadB* promoter is prevented by long chain fatty acyl coenzyme A. J. Biol. Chem. 267, 8685–8691. 1569108

[B11] Domínguez D. C. Guragain M. Patrauchan M. (2015). Calcium binding proteins and calcium signaling in prokaryotes. Cell Calcium 57, 151–165. 10.1016/j.ceca.2014.12.006 25555683

[B12] Epstein W. (2003). The roles and regulation of potassium in bacteria. Prog. Nucleic Acid Res. Mol. Biol. 75, 293–320. 10.1016/S0079-6603(03)75008-9 14604015

[B13] Fiedler S. Steinbüchel A. Rehm B. H. A. (2000). PhaG-mediated synthesis of poly(3-hydroxyalkanoates) consisting of medium-chain-length constituents from nonrelated carbon sources in recombinant *Pseudomonas fragi*. Appl. Environ. Microbiol. 66, 2117–2124. 10.1128/AEM.66.5.2117-2124.2000 10788390 PMC101463

[B14] Galbe M. Zacchi G. (2002). A review of the production of ethanol from softwood. Appl. Microbiol. Biotechnol. 59, 618–628. 10.1007/s00253-002-1058-9 12226717

[B15] Holland I. B. Jones H. E. Campbell A. K. Jacq A. (1999). An assessment of the role of intracellular free Ca^2+^ in E. coli. Biochimie 81, 901–907. 10.1016/S0300-9084(00)87175-8 10572304

[B16] Hood M. I. Skaar E. P. (2012). Nutritional immunity: transition metals at the pathogen-host interface. Nat. Rev. Microbiol. 10, 525–537. 10.1038/nrmicro2836 22796883 PMC3875331

[B17] Inbaraj B. S. Chen B.-H. (2012). *In vitro* removal of toxic heavy metals by poly(γ-glutamic acid)-coated superparamagnetic nanoparticles. Int. J. Nanomed. 7, 4419–4432. 10.2147/IJN.S34396 22927758 PMC3420602

[B18] Iram S. H. Cronan J. E. (2005). Unexpected functional diversity among FadR fatty acid transcriptional regulatory proteins. J. Biol. Chem. 280, 32148–32156. 10.1074/jbc.M504054200 16027119

[B19] Kiemle D. J. Stipanovic A. J. Mayo K. E. (2003). Proton NMR methods in the compositional characterization of polysaccharides, in Hemicelluloses: Science and Technology, ACS Symposium Series (Washington, DC: American Chemical Society), 122–139. 10.1021/bk-2004-0864.ch009

[B20] Kung C. Martinac B. Sukharev S. (2010). Mechanosensitive channels in microbes. Annu. Rev. Microbiol. 64, 313–329. 10.1146/annurev.micro.112408.134106 20825352

[B21] Laurijssen J. Marsidi M. Westenbroek A. Worrell E. Faaij A. (2010). Paper and biomass for energy?: the impact of paper recycling on energy and CO_2_ emissions. Resour. Conser. Recycl. 54, 1208–1218. 10.1016/j.resconrec.2010.03.016

[B22] Laycock B. Halley P. Pratt S. Werker A. Lant P. (2013). The chemomechanical properties of microbial polyhydroxyalkanoates. Prog. Polym. Sci. 38, 536–583. 10.1016/j.progpolymsci.2012.06.003

[B23] Lee S. Y. (1996). Bacterial polyhydroxyalkanoates. Biotechnol. Bioeng. 49, 1–14. 10.1002/(SICI)1097-0290(19960105)49:1<1::AID-BIT1>3.0.CO;2-P 18623547

[B24] Lessard I. A. Pratt S. D. McCafferty D. G. Bussiere D. E. Hutchins C. Wanner B. L.. (1998). Homologs of the vancomycin resistance D-Ala-D-Ala dipeptidase VanX in *Streptomyces toyocaensis, Escherichia coli* and *Synechocystis*: attributes of catalytic efficiency, stereoselectivity and regulation with implications for function. Chem. Biol. 5, 489–504. 10.1016/S1074-5521(98)90005-9 9751644

[B25] Li S. J. Cronan J. E. (1993). Growth rate regulation of *Escherichia coli* acetyl coenzyme A carboxylase, which catalyzes the first committed step of lipid biosynthesis. J. Bacteriol. 175, 332–340. 10.1128/jb.175.2.332-340.1993 7678242 PMC196146

[B26] Lu J. Tappel R. C. Nomura C. T. (2009). Mini-review: biosynthesis of poly(hydroxyalkanoates). Polymer Rev. 49, 226–248. 10.1080/15583720903048243

[B27] Martins A. Machado L. Costa S. Cerca P. Spengler G. Viveiros M.. (2011). Role of calcium in the efflux system of *Escherichia coli*. Int. J. Antimicrob. Agents 37, 410–414. 10.1016/j.ijantimicag.2011.01.010 21419607

[B28] Matsusaki H. Abe H. Doi Y. (2000). Biosynthesis and properties of poly(3-hydroxybutyrate-co-3-hydroxyalkanoates) by recombinant strains of *Pseudomonas* sp. 61-3. Biomacromolecules 1, 17–22. 10.1021/bm9900040 11709837

[B29] Min B. C. Bhayani B. V. Jampana V. S. Ramarao B. V. (2015). Enhancement of the enzymatic hydrolysis of fines from recycled paper mill waste rejects. Bioresour. Bioprocess. 2:40 10.1186/s40643-015-0068-2

[B30] Min B. C. Jampana S. N. Thomas C. M. Ramarao B. V. (2018). Study of buffer substitution using inhibitory compound CaCO3 in enzymatic hydrolysis of paper mill waste fines. J. Korea TAPPI 50, 77–82. 10.7584/JKTAPPI.2018.04.50.2.77

[B31] Min B. C. Ramarao B. V. (2017). Mechanisms of the inhibition of enzymatic hydrolysis of waste pulp fibers by calcium carbonate and the influence of nonionic surfactant for mitigation. Bioprocess Biosyst. Eng. 40, 799–806. 10.1007/s00449-017-1745-7 28197730

[B32] Mitra R. S. Gray R. H. Chin B. Bernstein I. A. (1975). Molecular mechanisms of accommodation in *Escherichia coli* to toxic levels of Cd^2+^. J. Bacteriol. 121, 1180–1188. 1090597 10.1128/jb.121.3.1180-1188.1975 PMC246051

[B33] Neumann M. Leimkühler S. (2008). Heavy metal ions inhibit molybdoenzyme activity by binding to the dithiolene moiety of molybdopterin in Escherichia coli. FEBS J. 275, 5678–5689. 10.1111/j.1742-4658.2008.06694.x 18959753

[B34] Noda I. Bond E. B. Green P. R. Melik D. H. Narasimhan K. Schechtman L. A. (2005). Preparation, properties, and utilization of biobased biodegradable Nodax^TM^ copolymers, in Polymer Biocatalysis and Biomaterials, ACS Symposium Series (Washington, DC: American Chemical Society), 280–291. 10.1021/bk-2005-0900.ch019

[B35] Nomura C. T. Tanaka T. Gan Z. Kuwabara K. Abe H. Takase K.. (2004). Effective enhancement of short-chain-length–medium-chain-length polyhydroxyalkanoate copolymer production by coexpression of genetically engineered 3-ketoacyl-acyl-carrier-protein synthase III (*fabH*) and polyhydroxyalkanoate synthesis genes. Biomacromolecules 5, 1457–1464. 10.1021/bm049959v 15244465

[B36] Norris V. Grant S. Freestone P. Canvin J. Sheikh F. N. Toth I.. (1996). Calcium signalling in bacteria. J. Bacteriol. 178, 3677–3682. 10.1128/jb.178.13.3677-3682.1996 8682765 PMC178146

[B37] Ogunleye A. Bhat A. Irorere V. U. Hill D. Williams C. Radecka I. (2015). Poly-γ-glutamic acid: production, properties and applications. Microbiology 161, 1–17. 10.1099/mic.0.081448-0 25288645

[B38] Peng L. Lifang R. Hongyu X. Xi L. Chaocan Z. (2007). Study on the toxic effect of lead(II) ion on *Escherichia coli*. Biol. Trace Elem. Res. 115, 195–202. 10.1007/BF02686030 17435262

[B39] Rehm B. H. A. Mitsky T. A. Steinbüchel A. (2001). Role of fatty acid *de novo* biosynthesis in polyhydroxyalkanoic acid (PHA) and rhamnolipid synthesis by Pseudomonads: establishment of the transacylase (PhaG)-mediated pathway for PHA biosynthesis in *Escherichia coli*. Appl. Environ. Microbiol. 67, 3102–3109. 10.1128/AEM.67.7.3102-3109.2001 11425728 PMC92987

[B40] Reusch R. N. Huang R. Bramble L. L. (1995). Poly-3-hydroxybutyrate/polyphosphate complexes form voltage-activated Ca^2+^ channels in the plasma membranes of *Escherichia coli*. Biophys. J. 69, 754–766. 10.1016/S0006-3495(95)79958-1 8519976 PMC1236305

[B41] Romani A. M. Scarpa A. (2000). Regulation of cellular magnesium. Front. Biosci. 5, D720–D734. 10.2741/A546 10922296

[B42] Saini S. Chutani P. Kumar P. Sharma K. K. (2019). Development of an eco-friendly deinking process for the production of bioethanol using diverse hazardous paper wastes. Renew. Energy 146, 2362–2373. 10.1016/j.renene.2019.08.087

[B43] Salamanca-Cardona L. Scheel R. A. Bergey N. S. Stipanovic A. J. Matsumoto K. Taguchi S.. (2016). Consolidated bioprocessing of poly(lactate-co-3-hydroxybutyrate) from xylan as a sole feedstock by genetically-engineered *Escherichia coli*. J. Biosci. Bioeng. 122, 406–414. 10.1016/j.jbiosc.2016.03.009 27067372

[B44] Salamanca-Cardona L. Scheel R. A. Lundgren B. R. Stipanovic A. J. Matsumoto K. Taguchi S.. (2014). Deletion of the *pflA* gene in *Escherichia coli* LS5218 and its effects on the production of polyhydroxyalkanoates using beechwood xylan as a feedstock. Bioengineered 5, 284–287. 10.4161/bioe.29595 25482228 PMC4156488

[B45] Sambrook J. J. Russell D. W. (2001). Molecular Cloning: A Laboratory Manual. Cold Spring Harbor, NY: Cold Spring Harbor Laboratory Press.

[B46] Scheel R. A. Ji L. Lundgren B. R. Nomura C. T. (2016). Enhancing poly(3-hydroxyalkanoate) production in *Escherichia coli* by the removal of the regulatory gene *arcA*. AMB Express 6:120. 10.1186/s13568-016-0291-z 27878786 PMC5120623

[B47] Shang L. Jiang M. Chang H. N. (2003). Poly(3-hydroxybutyrate) synthesis in fed-batch culture of *Ralstonia eutropha* with phosphate limitation under different glucose concentrations. Biotechnol. Lett. 25, 1415–1419. 10.1023/A:1025047410699 14514042

[B48] Shih I. L. Van Y. T. Yeh L. C. Lin H. G. Chang Y. N. (2001). Production of a biopolymer flocculant from *Bacillus licheniformis* and its flocculation properties. Bioresour. Technol. 78, 267–272. 10.1016/S0960-8524(01)00027-X 11341686

[B49] Siao F. Y. Lu J. F. Wang J. S. Inbaraj B. S. Chen B. H. (2009). *In vitro* binding of heavy metals by an edible biopolymer poly(γ-glutamic acid). J. Agric. Food Chem. 57, 777–784. 10.1021/jf803006r 19128012

[B50] Stephanopoulos G. Aristidou A. A. Nielsen J. (1998). Metabolic Engineering: Principles and Methodologies, 1st Edn. San Diego, CA: Academic Press.

[B51] Sudesh K. Abe H. Doi Y. (2000). Synthesis, structure and properties of polyhydroxyalkanoates: biological polyesters. Prog. Polym. Sci. 25, 1503–1555. 10.1016/S0079-6700(00)00035-6

[B52] Takamura Y. Nomura G. (1988). Changes in the intracellular concentration of acetyl-CoA and malonyl-CoA in relation to the carbon and energy metabolism of *Escherichia coli* K12. J. Gen. Microbiol. 134, 2249–2253. 10.1099/00221287-134-8-2249 3075658

[B53] Tappel R. C. Kucharski J. M. Mastroianni J. M. Stipanovic A. J. Nomura C. T. (2012). Biosynthesis of poly[(R)-3-hydroxyalkanoate] copolymers with controlled repeating unit compositions and physical properties. Biomacromolecules 13, 2964–2972. 10.1021/bm301043t 22873826

[B54] Tappel R. C. Pan W. Bergey N. S. Wang Q. Patterson I. L. Ozumba O. A. (2014). Engineering *Escherichia coli* for improved production of short-chain-length-*co-*medium-chain-length poly[(*R*)-3-hydroxyalkanoate] (SCL-*co*-MCL PHA) copolymers from renewable nonfatty acid feedstocks. ACS Sustain. Chem. Eng. 2, 1879–1887. 10.1021/sc500217p

[B55] Villanueva A. Wenzel H. (2007). Paper waste – recycling, incineration or landfilling? A review of existing life cycle assessments. Waste Manage. 27, S29–S46. 10.1016/j.wasman.2007.02.019 17433657

[B56] Wang L. Sharifzadeh M. Templer R. Murphy R. J. (2013). Bioethanol production from various waste papers: economic feasibility and sensitivity analysis. Appl. Energy 111, 1172–1182. 10.1016/j.apenergy.2012.08.048

[B57] Wang Q. Tappel R. C. Zhu C. Nomura C. T. (2012). Development of a new strategy for production of medium-chain-length polyhydroxyalkanoates by recombinant *Escherichia coli* via inexpensive non-fatty acid feedstocks. Appl. Environ. Microbiol. 78, 519–527. 10.1128/AEM.07020-11 22101037 PMC3255744

[B58] Wang Q. Yu H. Xia Y. Kang Z. Qi Q. (2009). Complete PHB mobilization in *Escherichia coli* enhances the stress tolerance: a potential biotechnological application. Microb. Cell Fact. 8:47. 10.1186/1475-2859-8-47 19719845 PMC2746179

[B59] Wei X. Ji Z. Chen S. (2010). Isolation of halotolerant *Bacillus licheniformis* WX-02 and regulatory effects of sodium chloride on yield and molecular sizes of poly-γ-glutamic acid. Appl. Biochem. Biotechnol. 160, 1332–1340. 10.1007/s12010-009-8681-1 19504190

[B60] Ye H. Jin L. Hu R. Yi Z. Li J. Wu Y.. (2006). Poly(gamma,L-glutamic acid)-cisplatin conjugate effectively inhibits human breast tumor xenografted in nude mice. Biomaterials 27, 5958–5965. 10.1016/j.biomaterials.2006.08.016 16949149

[B61] Yokoi H. Arima T. Hirose J. Hayashi S. Takasaki Y. (1996). Flocculation properties of poly(γ-glutamic acid) produced by *Bacillus subtilis*. J. Ferment. Bioeng. 82, 84–87. 10.1016/0922-338X(96)89461-X

[B62] Zhang Y.-H. P. (2008). Reviving the carbohydrate economy via multi-product lignocellulose biorefineries. J. Ind. Microbiol. Biotechnol. 35, 367–375. 10.1007/s10295-007-0293-6 18180967

