# Supplementary material for: Increased Production of the Value-Added Biopolymers Poly(R-3-Hydroxyalkanoate) and Poly(γ-Glutamic Acid) From Hydrolyzed Paper Recycling Waste Fines
Source: Front Bioeng Biotechnol. 2019 Dec 18;7:409. doi: 10.3389/fbioe.2019.00409 (PMC6930151; doi:10.3389/fbioe.2019.00409)
Supplement: Supplementary file 1 [file Table_1.DOCX]

Supplementary Material

# Supplementary Data

NMR spectra were recorded on a Bruker AVANCE III 600 MHz instrument, and were calibrated using residual undeuterated solvents as internal reference for PGA (water, δ = 4.79 ppm, ^1^H NMR). Chemical shifts (δ) are reported in parts per million (ppm); NMR peak multiplicities are denoted by the following abbreviations, when applicable: s = singlet, d = doublet, t = triplet, q = quartet, p = pentet, sext = sextet, dd = doublet of doublets, dt = doublet of triplets, m = multiplet, br = broad. Spectra were processed with Bruker TopSpin v3.5pI2.

## Supplementary Figures


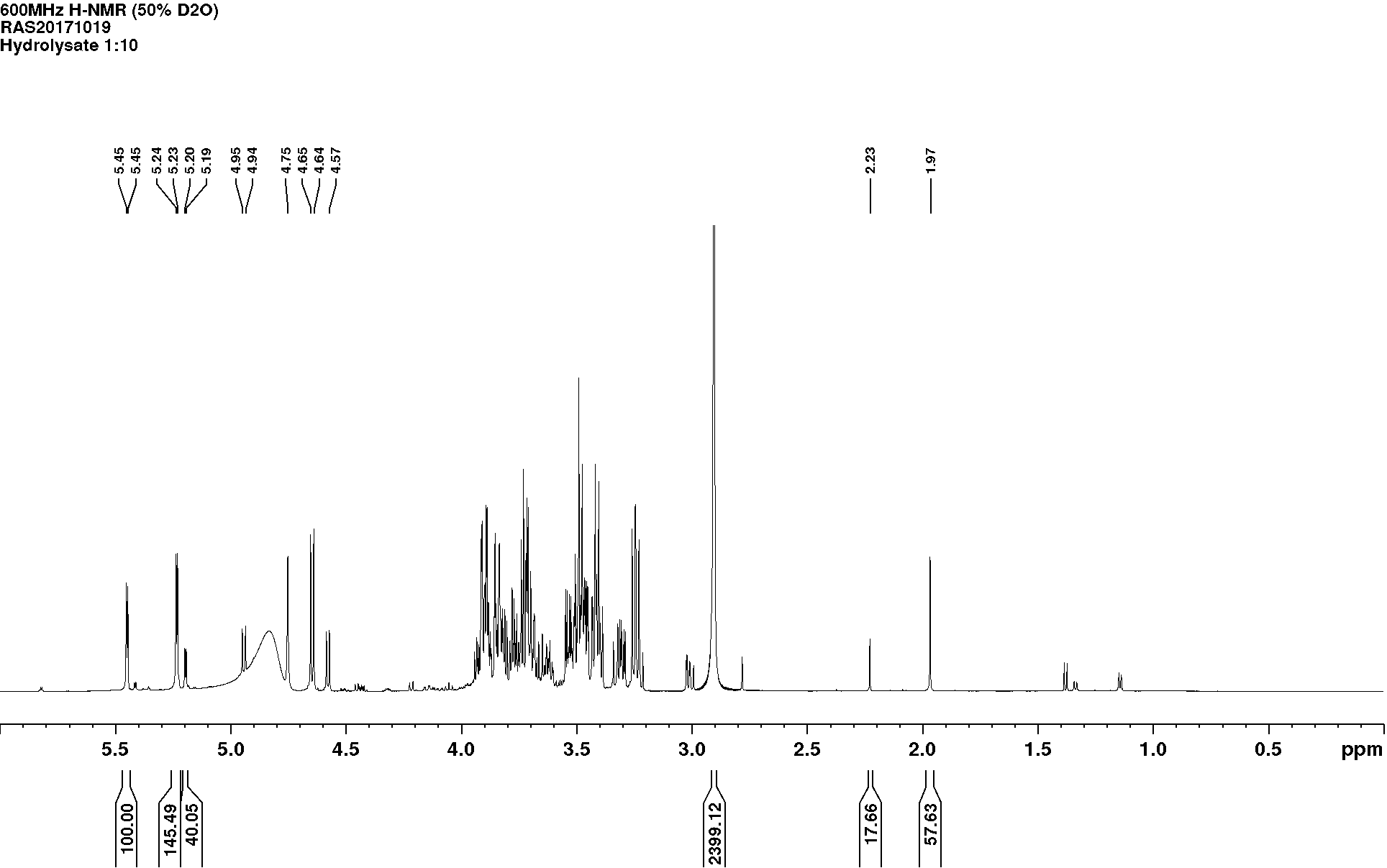


**Supplementary Figure 1:** ^1^H-NMR (600 MHz, 50% D_2_O) of crude hydrolysate in a 1:10 dilution with glucosamine as an internal standard; δ 5.45 (d, glucosamine α-anomeric H), δ 5.24-5.23 (d, glucose α-anomeric H), δ 5.20-5.19 (d, xylose α-anomeric proton), δ 4.95-4.94 (d, glucosamine β-anomeric H), δ 4.65-4.64 (d, glucose β-anomeric H), δ 4.57 (d, xylose β-anomeric H), δ 1.97 (s, 3H from acetate).


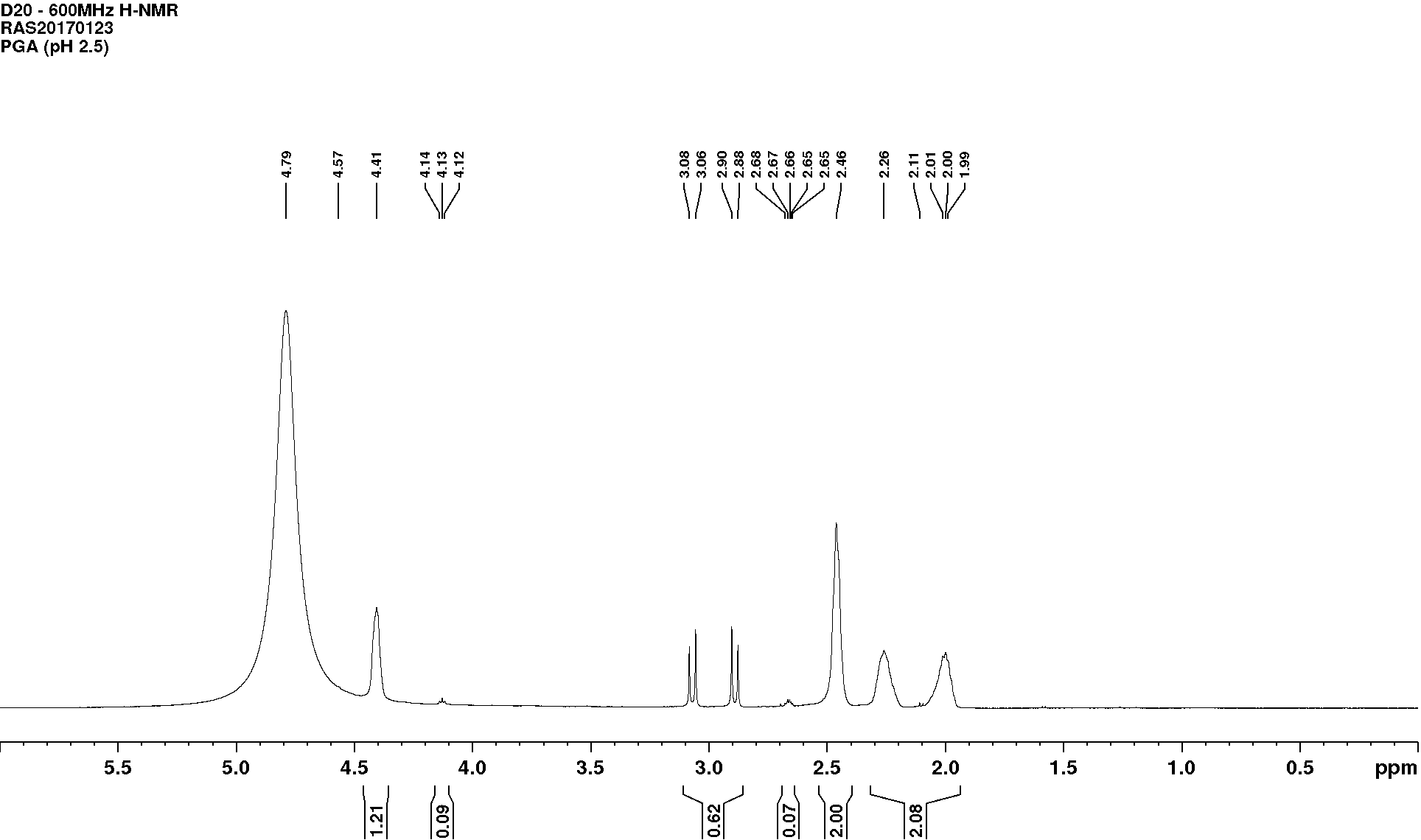


**PGA**


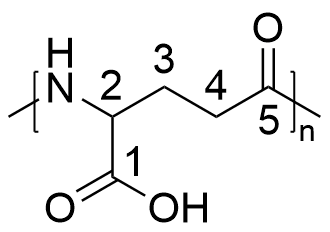


2

3

4

**Supplementary Figure 2:** ^1^H-NMR (600 MHz, D_2_O) of poly(γ-glutamic acid) (PGA); δ 4.41 (m, 1H), δ 3.08-2.88 (dd, unknown H), δ 2.46 (m, 2H), δ 2.11-1.99 (doublet of broad multiplets, 2H).

**Supplementary Figure 3:** COSY spectra (600 MHz, D_2_O) of poly(γ-glutamic acid). Determined that the unknown shift at δ 3.08-2.88 (Supplementary Figure 2) was not connected to the polymer.
